# Modeling global geometric spatial information for rotation invariant classification of satellite images

**DOI:** 10.1371/journal.pone.0219833

**Published:** 2019-07-19

**Authors:** Nouman Ali, Bushra Zafar, Muhammad Kashif Iqbal, Muhammad Sajid, Muhammad Yamin Younis, Saadat Hanif Dar, Muhammad Tariq Mahmood, Ik Hyun Lee

**Affiliations:** 1 Department of Software Engineering, Mirpur University of Science & Technology (MUST), Mirpur AJK, Pakistan; 2 Department of Computer Science, National Textile University, Faisalabad, Pakistan; 3 Department of Computer Science, Government College University, Faisalabad, Pakistan; 4 Department of Mathematics, Government College University, Faisalabad, Pakistan; 5 Department of Electrical Engineering, Mirpur University of Science & Technology (MUST), Mirpur AJK, Pakistan; 6 Department of Mechanical Engineering, Mirpur University of Science & Technology (MUST), Mirpur AJK, Pakistan; 7 School of Computer Science and Engineering, Korea University of Technology and Education, Cheonan, South Korea; 8 Department of Mechatronics, Korea Polytechnic University, Siheung-si, Gyeonggi-do, South Korea; Northeastern University, UNITED STATES

## Abstract

The classification of high-resolution satellite images is an open research problem for computer vision research community. In last few decades, the Bag of Visual Word (BoVW) model has been used for the classification of satellite images. In BoVW model, an orderless histogram of visual words without any spatial information is used as image signature. The performance of BoVW model suffers due to this orderless nature and addition of spatial clues are reported beneficial for scene and geographical classification of images. Most of the image representations that can compute image spatial information as are not invariant to rotations. A rotation invariant image representation is considered as one of the main requirement for satellite image classification. This paper presents a novel approach that computes the spatial clues for the histograms of BoVW model that is robust to the image rotations. The spatial clues are calculated by computing the histograms of orthogonal vectors. This is achieved by calculating the magnitude of orthogonal vectors between Pairs of Identical Visual Words (PIVW) relative to the geometric center of an image. The comparative analysis is performed with recently proposed research to obtain the best spatial feature representation for the satellite imagery. We evaluated the proposed research for image classification using three standard image benchmarks of remote sensing. The results and comparisons conducted to evaluate this research show that the proposed approach performs better in terms of classification accuracy for a variety of datasets based on satellite images.

## 1 Introduction

The classification of satellite images can facilities the factors that are management of environment, monitoring of earth, analysis of spatial data and outdoor and indoor mapping [1, 2]. The applications of remote sensing include the areas such as urban planning, agriculture, resource management and mineralogy [3, 4]. The main aim of remote sensing scene classification is to analyse the image spatial contents and assign a land-use category [5]. With the recent development in remote sensing technology, high resolution images can be obtained by using satellite and aircrafts and more useful spatial information can be extracted from these images [6]. The classification of remote sensing images is considered as a challenging task, as similar land sub-regions and low-level visual features are in different classes of image. There exists a significant research gap between high-level semantics and low-level feature-based image representation [5, 7, 8].

According to literature [9, 10], the classification of scene images is broadly divided into three categories that are based on the extraction of low-level, mid-level and high-level feature vectors. Color, texture and shape-based descriptors are the examples of low-level visual features and they are reported robust in case of uniform structures with very less variations in spatial contents [11]. Images with high resolution, diversity and non-homogenous spatial layout are difficult to analyse by using these low-level visual features [11, 12]. The mid-level feature representation is based on the development of image representation through the statical analysis of local features [11]. The Bag-of-Visual-Word (BoVW) model is one of the example of approaches that are based on mid-level feature representation. The final image signature for BoVW-based image representation contains no spatial clues that how visual words are arranged in the image plane [13, 14]. Various approaches in the literature are proposed to overcome the orderless nature of BoVW histogram-based representation [13]. Latent Dirichlet Allocation (LDA) and Probabilistic Latent Semantic Analysis (PLSA) are the examples of models that are developed to compute the spatial arrangement of features for scene analysis [15]. The approaches based on deep neural networks are the examples of high-level methods that can use multi-layers to learn image features [6]. The pre-trained neural networks can be retrained on new classes for scene classification and retrieval-based problems. The computational cost for training a large number of training samples is considered as the basic requirements to train deep networks [15].

The BoVW model has shown remarkable results in the fields such as text analysis, scene classification, object recognition and domains like image retrieval [16]. There are three main steps in BoVW model: feature extraction, vector quantization and histogram based image representation. One of the drawback associated with the BoVW approach is the lack of spatial information, which adversely affects the performance of remote sensing and scene classification [17–20]. Many extensions have been proposed in order to address this limitation [21]. According to the literature, Spatial Pyramid Matching (SPM) is one the popular technique that can compute the image spatial clues [21]. Yang *et al*. [22] analyzed the performance of BoVW and proposed two spatial variants for large-scale satellite image classification. The study demonstrated that the BoVW model provides comparable results against the state-of-the-art approaches. A major drawback reported is that the absolute spatial information captured by SPM degrades the classification performance for land use imagery. This is attributed to the fact that the land-use images contain significant rotations and rotation invariant image representation can enhance the classification accuracy. Yang *et al*. [23] propose Spatial Pyramid Co-occurrence Kernel (SPCK) that can capture the photometric and geometric aspects of images.

The relative spatial orientation of visual words often becomes the key discriminating information for imagery captured from satellite or aircraft [23]. Khan *et al*. [24] proposed PIWAH-based histogram for BoVW model, and computed the spatial clues by considering the visual words that are identical with respect to each other. However, the proposed approach is not invariant to rotation [24, 25]. A rotation invariant image representation is considered as one of the main requirement for remote sensing images [26, 27]. Anwar *et al*. [25] extended the PIWAH representation and proposed rotation invariant Triplets of Visual Words Angle Histogram (TIWAH) by computing the geometric relationships between triplets of identical visual words. Zafar *et al*. [28] presented Relative Geometric Spatial Image Representation (RGSIR), to enhance the classification and retrieval accuracy by computing global relative spatial orientation of visual words. Chen *et al*. [29] propose a rotation and translation invariant, Pyramid of Spatial Relatons (PSR), that combines the absolute and relative spatial information from images.

In this paper, we aim to extend the research of Khan *et al*. [24], by exploring Pairs Orthogonal Vector Histogram (POVH). The proposed image representation can compute the discriminative spatial clues and represent them in the histogram that is robust to image rotations. This is established by modeling the geometric relationships between Pairs of Identical Visual Words (PIVW) relative to the geometric center of an image (PIVW is a set of visual word pairs of same type). Later on, the spatial distribution of words in an image is formulated as a histogram-based on magnitude of the orthogonal vectors formed by PIVW. In addition to this, we also performed a comparative analysis to obtain the best spatial feature representation to obtain the optimal performance. The research presented in this paper is evaluated by using state-of-the-art remote sensing image benchmarks.

**Fig 1:**
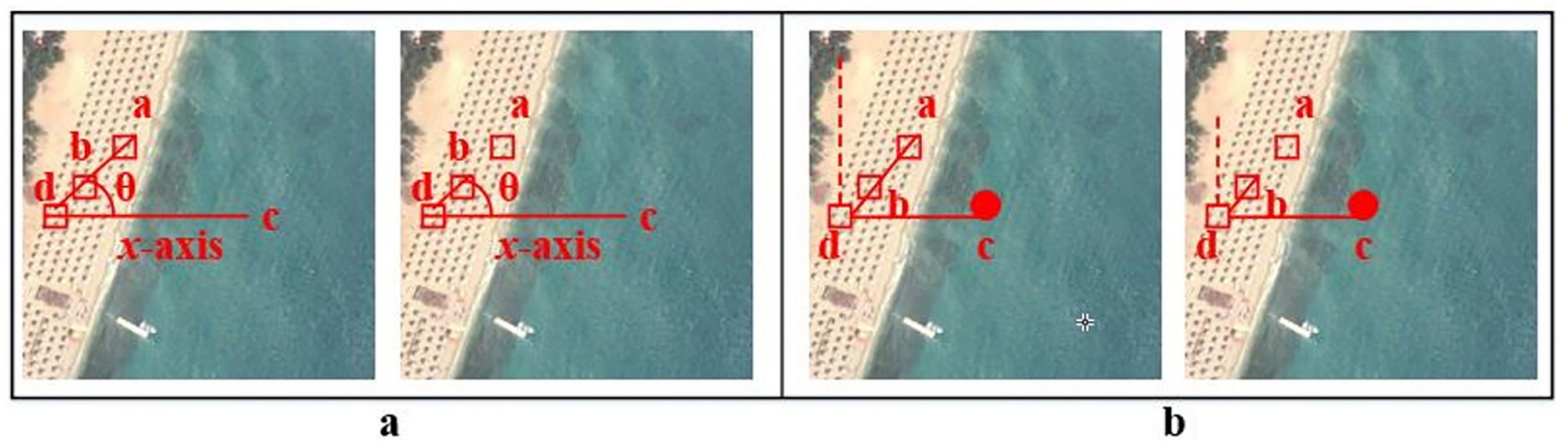
Collinear points (a) PIWAH [24] (b) POVH.

**Fig 2:**
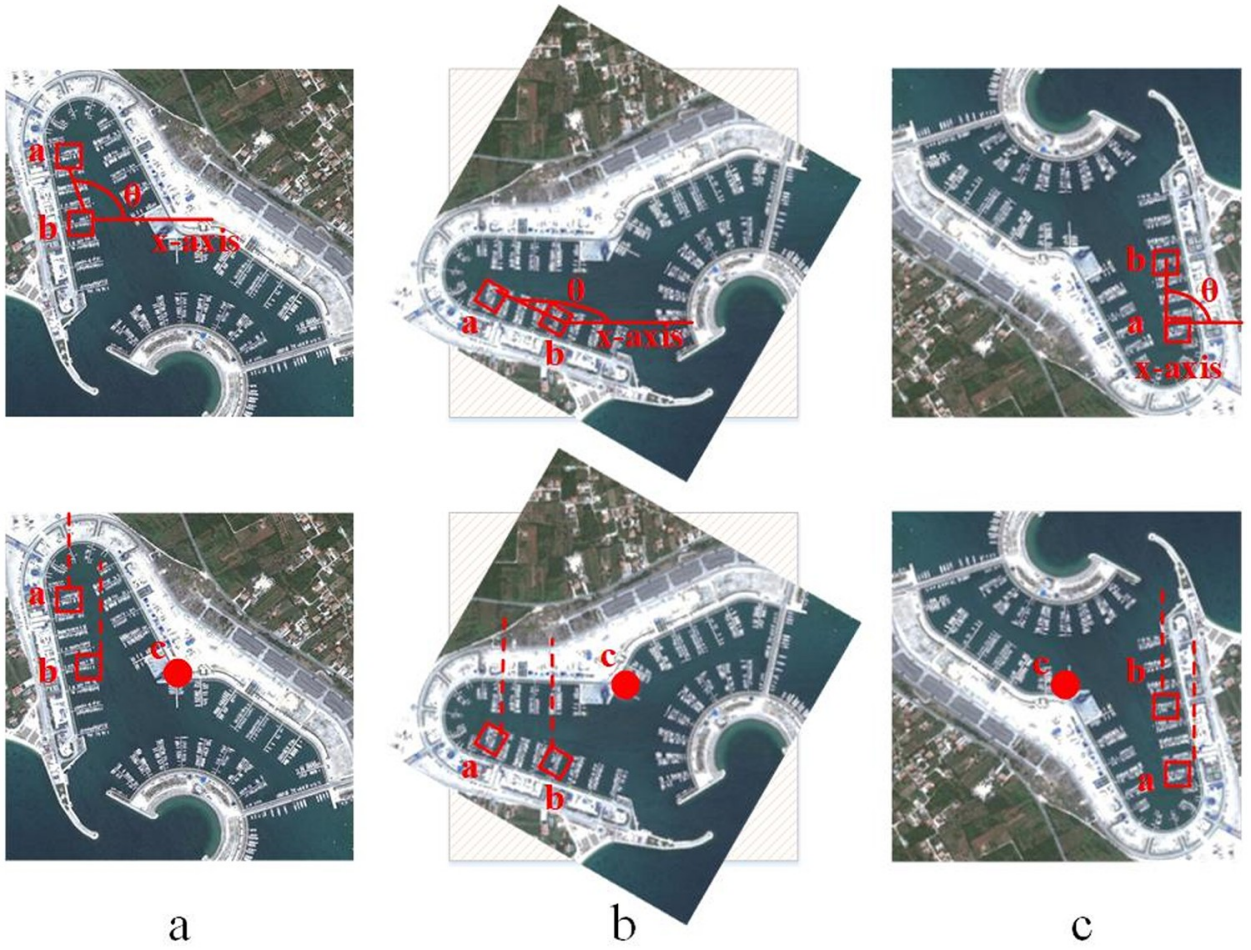
PIWAH [24] and POVH image representation (a) original image (b) image transformed by 60°rotation (c) image transformed by 180°rotation.

The proposed image representation is superior to the previous approaches in following aspects:

1. It computes the spatial clues for BoVW model provided by collinear points in images. The previous approaches [24, 25, 28] based on computation of angles between visual words are not able to capture the spatial clues provided by the collinear points in the images [13]. As it can be seen in Fig 1, that the points *a, b* and *d* are collinear as they lie on the same line. Fig 1(a) depicts the PIWAH [24] approach that loses the discriminative information provided by collinear points in images. The angles ∠*adc* and ∠*bdc* computed for these points are the same where *c* is the arbitrary point lying on the *x*-axis.
Fig 1(b) represents the proposed approach based on computation of magnitude of orthogonal vectors that are relative to the geometric center of an image. The magnitudes of orthogonal vectors $P_{d}^{ac}$ and $P_{d}^{bc}$ will be different. The proposed approach enriches the image representation with discriminative spatial clues thereby enhancing the predictive power of BOVW model.
2. The proposed representation based on PIVW, unlike PIWAH [24], is invariant to rotation. Being robust to rotation transformation is the main challenge for remotely sensing image classification [29]. Although TIWAH [25] is also invariant to image rotations, but it is worth mentioning here that if there are 20 identical words, then the total number of pair combinations will be 190 and the number of triplet combinations will be 1140. Our proposed approach significantly reduces the computational complexity.
  In Fig 2, the first row shows the PIWAH [24] image representation and the second row shows the proposed POVH approach. For both approaches (a) shows the original image, figures (b) and (c) shows the same image rotated at angle of 60° and 180°, respectively. Here we can see that different orientation information is provided by the same PIVW, for the original and rotated images. The ∠*abc* computed for the same PIVW will be different in the three cases. It is evident that the PIWAH [24] approach is not invariant to rotation.
  Whereas, the second row demonstrates the robustness of the proposed POVH representation. It can be seen that the magnitude of orthogonal vector for points *a* and *b* relative to the geometric center *c* is the same for the original and the rotated images, which proves that the proposed approach is invariant to rotation.
3. In addition to this, the proposed POVH representation is computationally efficient as it yields a low-dimensional image representation as compared to the complementary relative approaches.

The structure of the remaining paper is as: Section 2 is about recent state-of-the-art research. Section 3 is about the proposed methodology, that is about the proposed novel approach for the computation of histogram with image spatial clues, along with the details about feature extraction and experimental parameters. The image benchmarks and the discussion on image classification results are detailed in Section 4, while in Section 5 a discussion and sensitivity analysis of the proposed approach is presented. Section 6 provides conclusion and points towards the expected future direction of the proposed research.

## 2 Related work

This section is about a discussion of recent spatial feature extraction techniques, and other recently proposed approaches focused to enhance the classification accuracy for remote sensing scene classification. Aerial scene classification is considered as a challenging task as similar land sub-regions and low-level visual features are in the images that belongs to different categories [26, 27, 30, 31]. Xia *et al*. [32] presented a comprehensive review about the existing approaches that are used for classification of remote sensing images. They argued that the mid-level features can handle the variations caused by illumination changes, scale or rotation differences and provide a more compact vector representation for complex image structures and textures. The basic framework to create mid-level features representation involves extraction of local descriptors, such as: local texture, or spectral features, which are then aggregated using some encoding methods e.g., BoVW [33], Locality-constrained Linear Coding (LLC) [34], Probabilistic Latent Semantic Analysis (pLSA) [35], Improved Fisher kernel (IFK) [36] and Vector of Locally Aggregated Descriptors (VLAD) [37]. VLAD is a modified version of BoVW, as in addition to the feature distribution, it also computes the distance between the descriptors and the cluster centers. VLAD is reported to achieve better indexing accuracy as compared to the BoVW. Zafar *et al*. [38] propose to incorporate the absolute spatial information by computing weighed histograms of concentric circles.

The spatial relationships provide enormous and vast information for understanding and classifying images [39]. Lienou *et al*. proposed Latent Dirichlet Allocation (LDA) [40], a hierarchical model that creates image representation based on features extracted from a random selection of latent topics. Inspired from SPM, Ali *et al*. [16] proposed an absolute spatial feature extraction approach by computing histograms based on triangular regions in an image. The histograms hence created, captured the meaningful semantic information from different regions in images.

According to Zhu *et al*. [41], the classification performance of BoVW model for HSR images suffers due to the use of local features as they contain information about local patterns. Due to this reason, the authors proposed a hybrid image representation to improve the performance of BoVW model in terms of classification accuracy. The shape-based invariant texture features are computed to index the global texture information, while the standard deviation and mean values are used to compute the local spectral features. The structural features are also added by using dense Scale Invariant Feature Transform (SIFT) and the proposed feature fusion is reported efficient for remote sensing image classification. In another recent work, Ali *et al*. [42] demonstrated that the visual words integration of SIFT and SURF, adds the robustness of both features thereby enhancing the retrieval performance and reducing the semantic gap.

According to Feng *et al*. [43], the effective feature extraction and image representation is one the main requirement for classification-based problems. The authors present a Hybrid Histogram Descriptor (HHD) for satellite image matching application. The proposed hybrid representation is computed by using color and edge orientation and the motif co-occurrence histogram is calculated on the basis of motif patterns. Deng *et al*. [44] proposed an image representation that is based on discriminative models. Different distributions of heterogeneous features are computed to effectively combine the feature space to avoid the mismatch problem. The author proposed a semi supervised multiple kernel learning approach to generate the learning model from multi-feature space. The mismatch among the data is measured by using Multi-kernel Maximum Mean Discrepancy (MK-MMD).

Besides methods that are based on mid-level representation, the recent research is focused on analysis and comparison with deep-learning approaches [45–48]. Deep-Learning (DL) approaches have shown remarkable performance in diverse domains as image scene classification, healthcare, robot navigation systems and face recognition [49, 50]. Zhang *et al*. [51] proposed Saliency-Guided Unsupervised Feature Learning (S-UFL) scheme based on saliency detection algorithm. The authors demonstrated, that the statistics generated from S-UFL can improve the classification of complex scene images. Zou *et al*. [45] proposed a deep-learning based feature extraction method to boost the classification performance of high-resolution satellite images. The authors proposed an iterative algorithm, that directed the DBN to select reconstruction weights that could produce more discriminative reconstructible features. According to Gong *et al*. [46], low quality variance remained an open challenge for high resolution satellite imagery. The authors proposed a Deep Salient Feature based Anti-noise Transfer Network (DSFATN) approach to effectively explore the high-level image features for remote sensing images in varying scenarios of scale and noise.

According to Li *et al*. [47], the existing image representation techniques for remote sensing are based on image global features. The authors proposed deep features-based image representation that can extract global and region-based features that are inputs for pre-trained Convolutional neural network (CNN) model. The final feature vector is computed on the basis of regional deep-features with a modified approach based on VLAD. However, the CNNs are sensitive to the rotations that are in images and this can lead to misclassification of images. According to Liu *et al*. [48], the scale variations in images make scene classification a challenging task for remote sensing imagery. The authors proposed a multi-scale CNN to overcome the limitation. The high resolution satellite images exhibit diversity in spatial and structural patterns [51]. Kattan and Wei [52] performed a study to assess the effectiveness of CNN architecture i.e. Alexnet for remote sensing image benchmarks. AlexNet is a convolutional neural network that is trained on more than a million images from the ImageNet database [53]. The network is 8 layers deep and has learned rich feature representations for a wide range of images. Transfer Learning (TL) is a widely used approach in DL applications. In this method, a model developed for a task is reused as a starting point for other task [54]. It is much faster and convenient to fine-tune a network with TL, instead of training a network from the scratch with randomly initialized weights. The learned features can be easily transferred to a new task by using a smaller number of images. Data Augmentation (DA) scheme is used to boost CNN performance by artificially inflating the dataset to generate more invariant examples and avoid overfitting. In a comparative study, regarding performance evaluation of different DA schemes (i.e. cropping, rotation and flipping), the cropping scheme attained the highest performance [55]. This is because the cropping schemes generates more training samples as compared to other augmentation schemes thereby improving generalization, reducing overfitting and improving the overall classification performance. However, it is worth mentioning here that the approaches based on deep-learning are computationally expensive, and require large-scale data and time for training a classification-based model [13, 56]. Another disadvantage of using Neural Networks is that they have a “black-box” nature, meaning that the weights derived for each node, that contributes to a specific outcome, are not clearly justified [57]. Whereas the hand-crafted features are very interpretable. This is important because, in some domains, interpretability is quite important.

## 3 The proposed approach

The proposed research presented in this paper is based on the computation of orthogonal vectors relative to the geometric center of an image between PIVW’s. In addition, we performed a comparative analysis with different state-of-the-art spatial feature extraction techniques, to sort out the best spatial image representation for land-use scene classification. The proposed histogram representation and the implementation details are discussed in the following sub-sections.

### 3.1 Pairs Orthogonal Vector Histogram (POVH)

**Fig 3:**
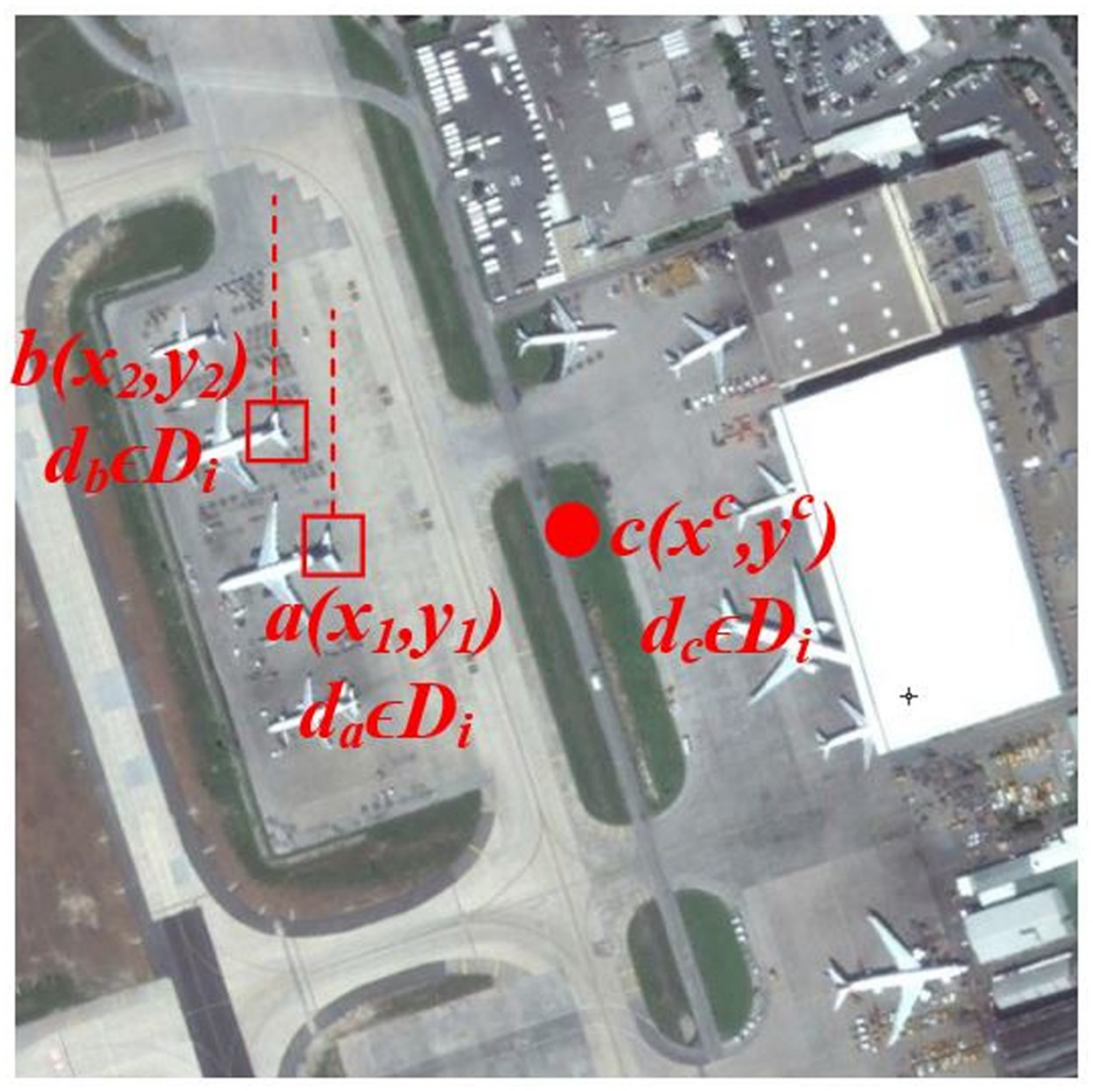
Spatial coordinate representation relative to the geometric center for identical visual words.

The proposed research is being evaluated by selecting the Bag-of-Visual-Words (BoVW) model. The main steps of the proposed method are described as:

1. In BoVW model, an image (*M*) is represented in the form of patches of local features that are computed as *M* = {*d*_1_, *d*_2_, *d*_3_, …, *d*_*K*_}, where *K* denotes the total number of descriptors.
2. The features extracted are in a high-dimensional feature space, that are quantized by applying *k*-means clustering into informative regions (termed as visual words), based on some distance measure. The visual vocabulary/codebook (which is a collection of visual words) is created, as *W* = *w*_1_, *w*_2_, *w*_3_, …*w*_*N*_, where *W* represents the codebook of size *N* with *N* clusters.
3. The descriptors are assigned to the nearest visual words as:
  $$\begin{array}{c} w\left(d_{i}\right)=arg\underset{w\in W}{min}\;Dist\left(w,d_{i}\right) \end{array}$$ (1)
  Here, *w*(*d*_*i*_) signifies the visual word assigned to the *i*^*th*^ descriptor, and the distance between *d*_*i*_ and *w* is given by *Dist*(*w*, *d*_*i*_).
4. Each image in the dataset is represented as a set of descriptors, where each descriptor is assigned to a particular cluster center/visual word from the codebook. The number of the histogram bins equate the count of the visual words in the codebook (i.e. *N*). If each histogram bin represents a visual word *w*_*i*_, in *W*, then
  $$\begin{array}{c} bin_{i}=card\left(D_{i}\right)\;\;where\;\;D_{i}=\left\{d_{j},j\in 1,.\ldots ,n|w\left(d_{j}\right)=w_{i}\right\} \end{array}$$ (2)
  where *D*_*i*_ is descriptor set related to a specific *w*_*i*_ in an image. *Card*(*D*_*i*_) is the cardinality that gives count of the elements of the set *D*_*i*_. To create the final histogram representation, this is repeated for every visual word in an image. The spatial information of interest points is not retained in this step.
5. Here to compute the POVH, we define the set of all PIVW’S related to a visual word *w*_*i*_ as:
  $$\begin{array}{c} PIVW_{i}=\left\{\left(a,b\right)|\left(d_{a},d_{b}\right)\in D_{i}^{2},d_{a}\neq d_{b}\right\} \end{array}$$ (3)
6. The cardinality of *PIVW*_*i*_ is ${}^{b_{i}}C_{2}$ that represents the total possible combinations that can exist between distinct vector pairs among *b*_*i*_ elements.
7. Given an image *M*, where the size of the image is *R* × *C*, the geometric center *c* = (*x*^*c*^, *y*^*c*^) is given by
  $$\begin{array}{c} x^{c}=\frac{1}{|M|}\sum_{(x,y)\in M}x,\;\;y^{c}=\frac{1}{|M|}\sum_{(x,y)\in M}y \end{array}$$ (4)
  where *M* = {(*x*, *y*)∣1 ≤ *x* ≤ *R*, 1 ≤ *y* ≤ *C*} and ∣*M*∣ denotes the total elements in *M*. Fig 3 gives an intuition to better understand the proposed approach.
8. The vectors $\vec{ac}$ and $\vec{ab}$ are given by:
  $$\begin{array}{c} \vec{ac}=\left(x^{c}-x_{1},y^{c}-y_{1}\right)\\ \vec{ab}=\left(x_{2}-x_{1},y_{2}-y_{1}\right) \end{array}$$
9. Let $P_{a}^{bc}$ represent the vector at *a* orthogonal to $\vec{ac}$ and $\vec{ab}$, then
  $$\begin{array}{cc} P_{a}^{bc} & =\vec{ac}\times \vec{ab}\\ & =|\begin{array}{cc} \hat{i} & \hat{j}\\ x^{c}-x_{1} & y^{c}-y_{1}\\ x_{2}-x_{1} & y_{2}-y_{1} \end{array}|\\ & =(\left(x^{c}-x_{1}\right)\left(y_{2}-y_{1}\right),\left(x_{1}-x_{2}\right)\left(y^{c}-y_{1}\right)) \end{array}$$
10. The magnitude of $P_{a}^{bc}$ is calculated as
  $$\begin{array}{c} |P_{a}^{bc}|=\sqrt{{\left[\left(x^{c}-x_{1}\right)\left(y_{2}-y_{1}\right)\right]}^{2}+{\left[\left(x_{1}-x_{2}\right)\left(y^{c}-y_{1}\right)\right]}^{2}} \end{array}$$ (5)
  Similarly, the magnitude of vector at b orthogonal to $\vec{ba}$ and $\vec{bc}$ can be calculated as
  $$\begin{array}{c} |P_{b}^{ac}|=\sqrt{{\left[\left(x_{1}-x_{2}\right)\left(y^{c}-y_{2}\right)\right]}^{2}+{\left[\left(x^{c}-x_{2}\right)\left(y_{2}-y_{1}\right)\right]}^{2}} \end{array}$$ (6)
11. The magnitude values obtained are scaled in the range of 0-1. POVH gives the spatial orientation of the visual word *w*_*i*_. The final image representation is created by concatenating POVH obtained from all visual words. The bins of the BoVW histogram are replaced by the corresponding *POV H*_*i*_, related to *w*_*i*_, by applying the bin replacement technique. The frequency information is kept intact by normalizing the sum of *POV H*_*i*_ bins to the bin-size *b*_*i*_ of the corresponding BoVW histogram bin that is being substituted. The POVH image representation is given by:
  $$\begin{array}{c} POV\;H=(\delta_{1}POV\;H_{1},\delta_{2}POV\;H_{2},‥\ldots ,\delta_{N}POV\;H_{N}) \end{array}$$ (7)
  where *δ*_*i*_ is the normalization coefficient and is given by $\delta_{i}=\frac{b_{i}}{||POV\;H_{i}\left|\right|}$. The dimensions of the resultant POVH feature vector are *N* × *H*, where N represents the vocabulary size and H shows the number of histogram bins.

### 3.2 Implementation details

**Fig 4:**
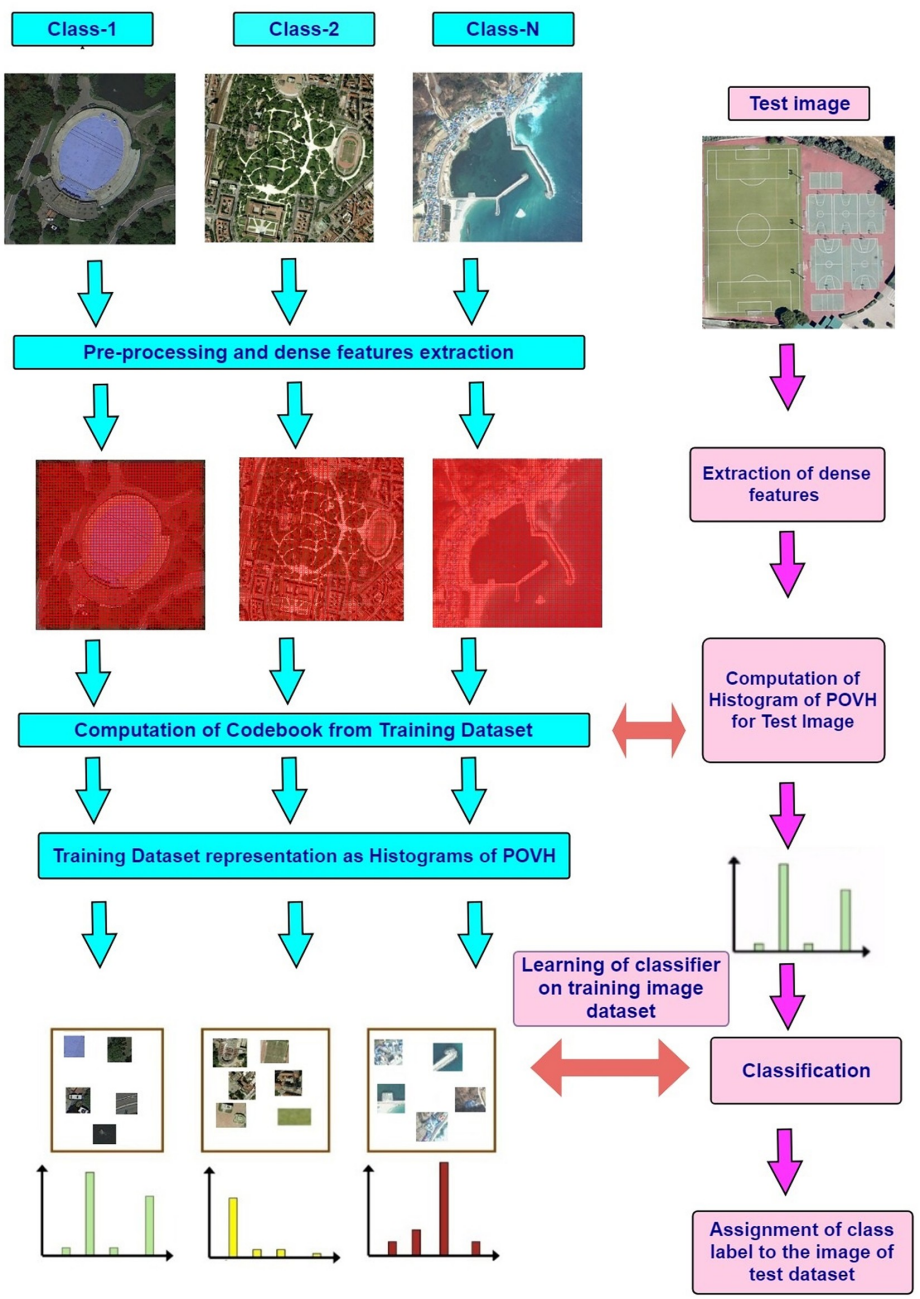
Block diagram of the proposed research model.

Fig 4 represents the main blocks of proposed research. It is important to mention here that for all datasets the same scheme/sequence of steps are followed to compute the final image representation. Initially all datasets are partitioned into two random sub-sets according to the specified training test ratio. One sub-set is used to training and the other is retained for testing. Then some necessary pre-processing tasks are carried out like converting images into gray-scale. As a part of this pre-processing the larger images are resized to 450 × 450 pixels to reduce the computational complexity that is associated with feature extraction and clustering.

To further reduce the computational complexity, 0.4% of random sub-set of keypoints are selected from the training dataset to create the codebook. In dense sampling [58], patches of fixed size and shape are placed on a regular grid. Dense sampling provides better coverage of the entire object or scene by computing a constant amount of features per image area. It provides the advantage that low contrast regions contribute equally to the overall image representation. For dense features we used a step size of 5 for each of image datasets and computed SIFT features after every 5th pixel. Histogram representations are then created based on the proposed POVH approach for the training sub-set of images. For testing sub-set, after feature extraction, histogram representations are created in accordance with the dictionary created for the training sub-set. The training histograms are used to train the classifier and the proposed approach is validated through histograms of test sub-sets. We applied a threshold and random selection to reduce the pairs between the same words. The results presented in the next section are based on 5-bins POVH representation.

Support Vector Machines [59] with Hellinger Kernel [60] is used for image classification. For comparative analysis, the base-line model BoVW and other spatial approaches as SPM [21], triangular histograms [16] and PIWAH [24] are executed in parallel, following the same experimental protocol as used for the proposed approach. All the experiments are repeated 10 trials to overcome the uncertainty due of unsupervised nature of *k*-means clustering. For each execution, we selected random images for training and testing and mean of values are reported in results.

In order to provide a comparison of the proposed approach with the state of art deep learning methods, experiments are performed with the AlexNet CNN architecture for the RSSCN and SIRI-WHU image datasets respectively. The AlexNet model has learned rich feature representations for a wide range of images. The early layers of the pre-trained network learned low-level features as edges, blobs and colors, whereas the last layers are based on task specific features. To reuse the pre-trained network, the final layers are replaced by new layers to learn dataset specific features. This enables faster learning with fewer classes. The network is then trained with training images to assess the prediction accuracy of network. We have used matlab implementation for experiments [61], for exact details on the Alexnet architecture, we refer the reader to [53, 62]. Our architecture only deviate from the architecture described there in the size of their final output layer.

## 4 Datasets and results

To evaluate the effectiveness of proposed research, experiments are conducted on benchmark datasets that are used extensively in the literature. We have selected these challenging datasets for two reasons. Firstly, as the proposed approach is a spatial feature extraction approach, spatial clues are very important for classification of high resolution remote sensing imagery. Secondly, the selected datasets are very diverse and exhibit significant orientation and scale variations. A description of each dataset is provided below:

### 4.1 Dataset description

**Fig 5:**
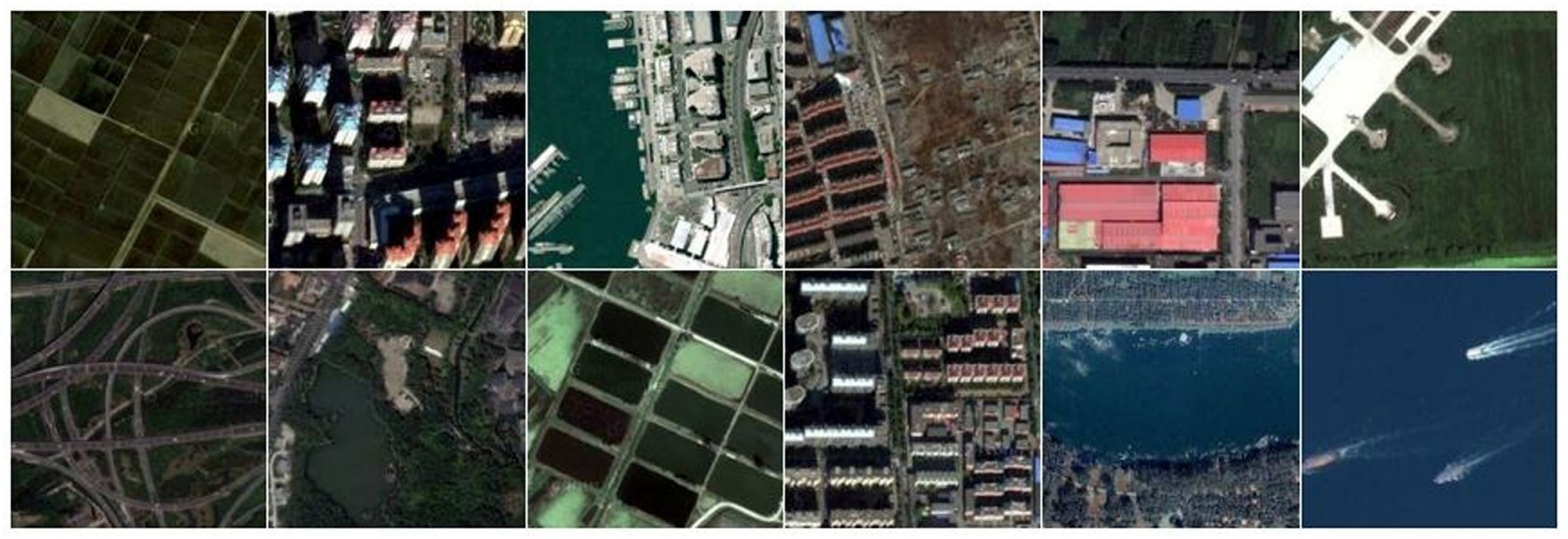
A photo gallery of images from SIRI-WHU dataset.

The first dataset used for the evaluation of the proposed POVH representation is the SIRI-WHU image dataset [63]. This Google image dataset covers the urban areas in China and is collected by the RSIDEA (Intelligent Data Extraction, Analysis and Applications of Remote Sensing) group, LIESMARS, Wuhan University. The Google SIRI-WHU image dataset comprises of 2400 images, classified into 12 categories with 200 images per-class. The images have a size of 200 × 200 pixels and a spatial resolution of 2-m. For all datasets, we used 70% stochastically selected images per-class as training samples, and the remaining are retained for testing. Fig 5 represents the photo gallery of images taken from SIRI-WHU dataset.

**Fig 6:**
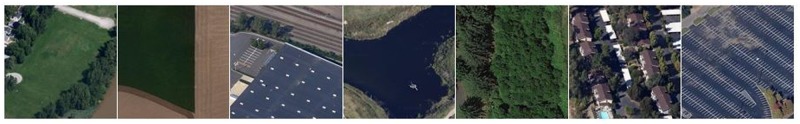
A photo gallery of images from RSSCN image dataset.

The second dataset used is our experiments is the RSSCN image dataset [45], released in 2015, comprising of images collected from Google Earth. It consists of 2800 images categorized into 7 typical scene categories. There are 400 images per-class, and each image has a size of 400 × 400 pixels. It is a challenging dataset as the images in each class are sampled at 4 different scales, with 100 images at per scale under varied imaging angles. Fig 6 represents the photo gallery of images taken from RSSCN dataset.

**Fig 7:**
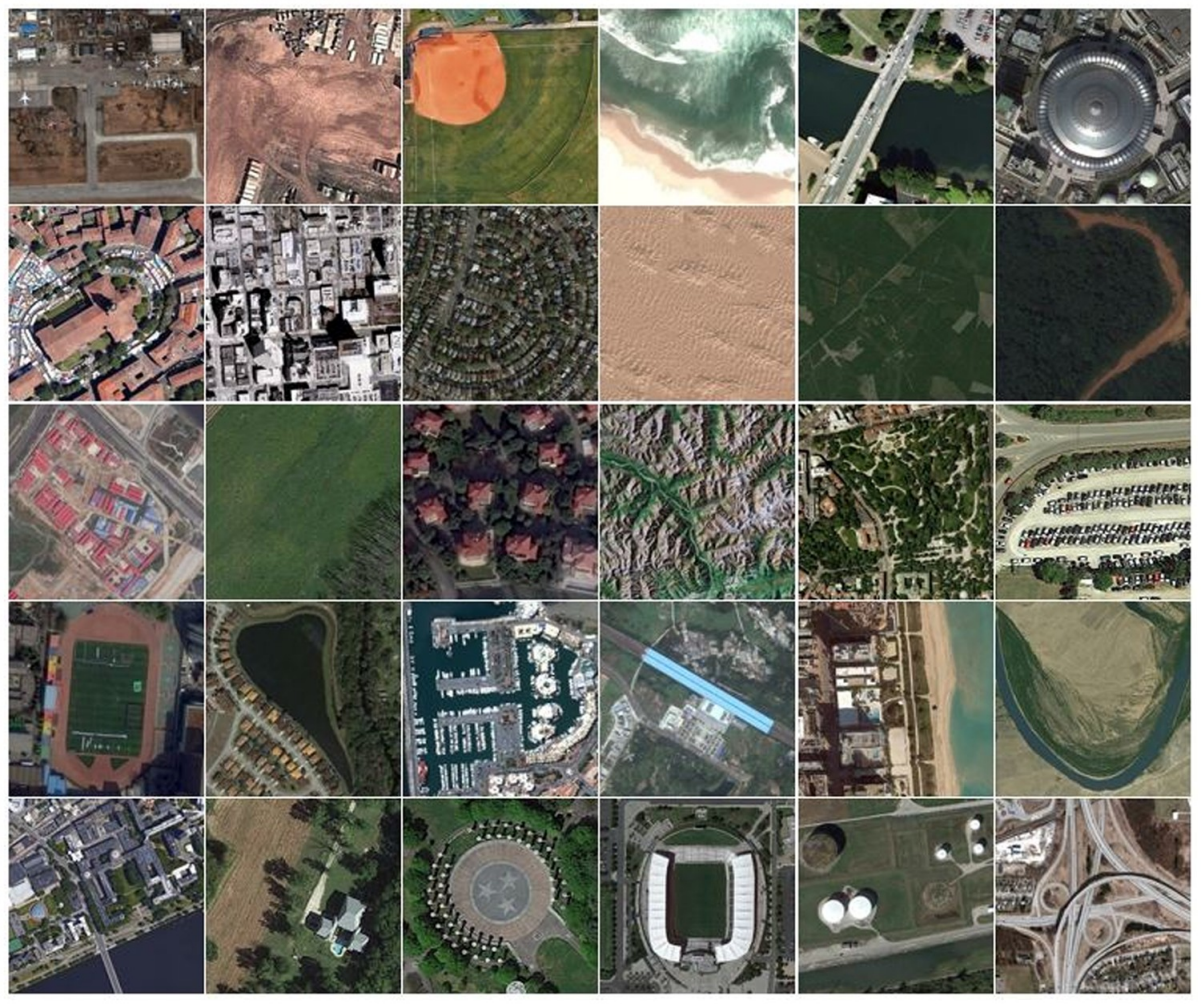
A photo gallery of images from AID image dataset.

The third is the recently introduced large-scale aerial image dataset (AID), comprising of images downloaded from Google Earth [64]. It consists of a total of 10,000 images organized into 30 categories. It is a challenging dataset as the Google Earth images are captured from different remote sensing sensors. It is multi-source and multi-resolution dataset with image size of about 600 × 600 pixels. In addition to this, it exhibits high intra-class diversity as the images are captured at different scales, orientations and imaging conditions. Fig 7 represents the photo gallery of images taken from AID image dataset.

### 4.2 Classification of SIRI-WHU image dataset

**Fig 8:**
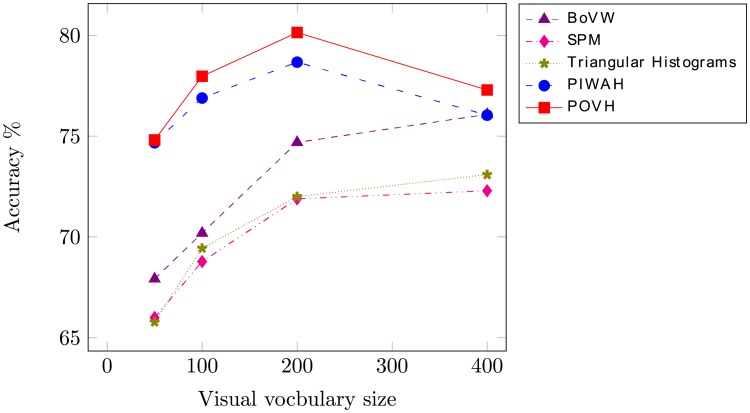
Representation of classification accuracy obtained when using vocabulary of different sizes for SIRI-WHU dataset.

To assess the effectiveness of the proposed approach, experiments are performed with the SIRI-WHU image dataset. Fig 8 provides an illustration of the performance of the proposed and the state-of-the-art spatial approaches for different sizes of visual vocabulary. The optimal performance for the proposed approach is obtained for a visual vocabulary size of 200. The dimensions of the resultant feature vector are 1000.

Table 1 provides a comparison of the classification performance and dimensions of the proposed approach with the state-of-the-art spatial feature extraction approaches. It can be seen evidently, that the proposed scheme POVH, outperforms the concurrent absolute and relative spatial feature extraction approaches, attaining the highest classification accuracy. The proposed approach provides 5.45% higher accuracy as compared to the conventional BoVW model. SPM and triangular histograms encode the absolute spatial information. The results are reported for level-1 SPM and 2 × 2 triangular regions. The proposed approach provides 8.25% and 8.14% higher accuracy compared to the mentioned absolute approaches. Another point of interest is the dimensions of the resultant feature vector. It can be seen that the dimensions of SPM and triangular histograms are 800. The dimensions of the proposed feature vector are 1000, that are $\frac{5}{4}$ of these absolute spatial approaches.

**Table 1:** Classification accuracy and size of feature vector comparison while using SIRI-WHU dataset.

Algorithms	Feature Dimensionality	Accuracy
BoVW	200	74.69%
SPM	800	71.89%
Triangular Histograms	800	72%
PIWAH	1800	78.67%
POVH	1000	80.14%

Next, the proposed POVH is compared to the relative spatial feature technique i.e. PIWAH. The proposed approach outperforms PIWAH by providing 1.47% higher classification accuracy. The dimensions of the resultant feature vector are 1000, which is 0.8 times less as compared to PIWAH and the proposed image representation. Experimental results demonstrate the effectiveness of the proposed approach in recognizing the complex remote scene image categories.

Table 2 provides a comparison of the proposed approach with the state-of-the-art approaches. The proposed approach provides 13.29% higher accuracy as compared to LDA, and 9.25% higher performance than LLC. The proposed POVH outperforms S-UFL, TL and CNN (6conv+2fc) by 5.3%, 3.9% and 1.94% respectively. The proposed image representation achieves the best performance as compared to the state-of-the-art approaches.

**Table 2:** Comparison of classification accuracy while using SIRI-WHU dataset.

Algorithms	Accuracy
LDA [65]	66.85%
LLC [66]	70.89%
S-UFL [66]	74.84%
TL [61]	76.2%
CNN(6conv+2fc) [46]	78.20
POVH	80.14%

**Fig 9:**
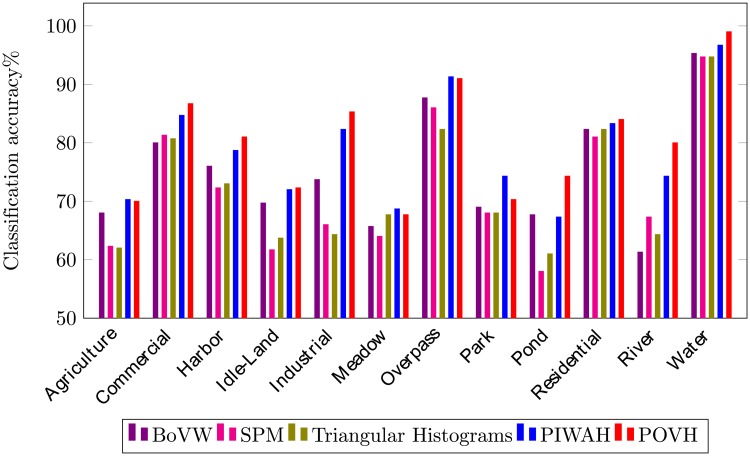
Class-wise comparison for SIRI-WHU dataset.

The class-wise comparison (in terms of classification accuracy), obtained from the proposed research and other spatial image representations is presented in Fig 9. Our proposed approach shows a remarkable performance by correctly classifying images into their semantic categories. Even the complex classes such as: overpass and idle-land, and river and harbor sharing same structural and spectral features, show better classification score while using the proposed POVH. The POVH successfully captures the discriminative spatial features from complex images thereby providing the highest accuracy.

### 4.2 Classification of RSSCN image dataset

**Fig 10:**
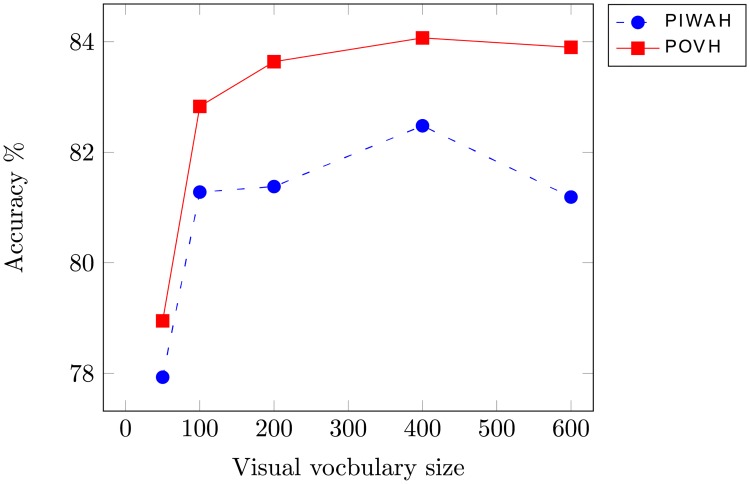
Comparison of classification accuracy with the research based on spatial approaches for RSSCN dataset.

To obtain the optimal performance for the proposed the proposed image representation, experiments are performed with different sizes of visual vocabulary. As it can be seen in Fig 10, the best performance for POVH is obtained for a visual vocabulary size of 400, resulting in a 2000 dimensional feature vector. As the proposed approach is inspired from the PIWAH [24] approach, Table 3 provides a comparison of the proposed image representation with PIWAH in terms of accuracy and feature dimensionality. It is important to note here, that PIWAH and the proposed POVH are relative spatial feature techniques. The proposed approach outperforms PIWAH by providing 1.59% higher classification accuracy. The dimensions of resultant feature vector are 2000, which are $\frac{5}{9}$ of PIWAH. The proposed image representation not only outperforms the state-of-the-art absolute and relative spatial feature extraction approaches in terms of classification accuracy but also significantly reduces the dimensions of the PIWAH feature vector.

**Table 3:** Classification accuracy and size of feature vector comparison while using RSSCN image dataset.

Algorithms	Feature Dimensionality	Accuracy
PIWAH	3600	82.48%
POVH	2000	84.07%

The results presented in Table 4 demonstrate that the proposed scheme for image spatial representation outperforms the state-of-the-art mid-level approaches. It can be evidently seen that the proposed approach achieves 10.21% higher accuracy as compared to LDA (SIFT), and 7.07% higher classification performance than the deep-learning approach used by the creator of the dataset. The proposed approach outperforms TL, LLC (CH), pLSA (SIFT), VLAD (SIFT) and RGSIR by 10.87%, 4.13%, 4.7%, 4.73% and 3.07% respectively. The proposed POVH approach provides promising results by maintaining consistent performance on complex image benchmarks.

**Table 4:** Classification accuracy comparison while using RSSCN dataset.

Algorithms	Accuracy
TL [61]	73.2%
LDA (SIFT) [64]	73.86%
Zou *et al*. [45]	77%
LLC (CH) [64]	79.94%
pLSA (SIFT) [64]	79.37%
VLAD (SIFT) [64]	79.34%
RGSIR [28]	81%
POVH	84.07%

**Fig 11:**
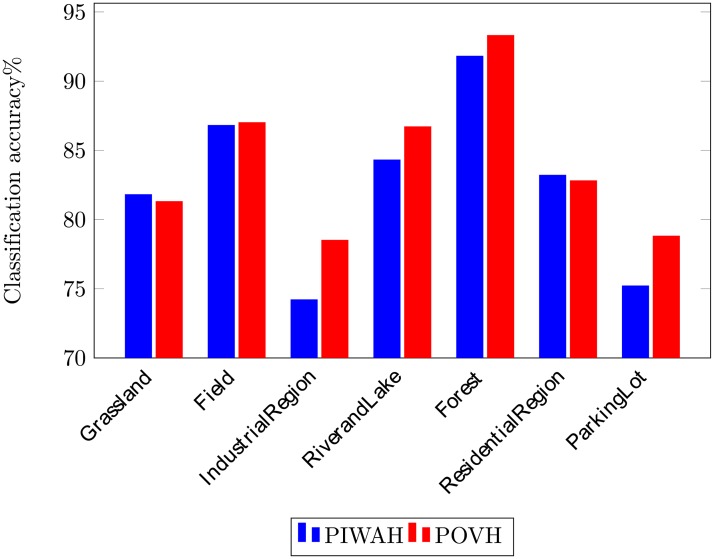
Class-wise comparison while using RSSCN dataset.

Fig 11 provides class-wise comparison of the proposed approach with the spatial feature extraction approaches. The comparison clearly demonstrates the superiority of the proposed POVH to the concurrent relative spatial feature extraction approach. The POVH image representation successfully classifies images into their respective semantic classes.

### 4.4 Classification of AID image dataset

To further evaluate the robustness of the proposed approach, experiments are conducted on the AID image dataset. Table 5 provides a comparison of the proposed approach with the state-of-the-art mid-level classification approaches. The proposed approach provides 7.72% higher accuracy as compared to the conventional BoVW model. The proposed approach achieves 25.28% and 12.85% better performance as compared to LDA and LLC respectively. The POVH outperforms the SPM and VLAD by 30.57% and 7.13% respectively. The proposed approach yields 5.71% higher accuracy as compared to the results reported by Kattan and Wei [52] for input size of 224. The experimental results validate the effectiveness of proposed approach for classification of remote sensing images.

**Table 5:** Classification accuracy comparison while using AID dataset.

Algorithms	Accuracy
BoVW (SIFT) [64]	68.37%
LDA (SIFT) [64]	50.81%
LLC (SIFT) [64]	63.24%
pLSA (SIFT) [64]	63.07%
SPM (SIFT) [64]	45.52%
VLAD (SIFT) [64]	68.96%
Kattan and Wei [52]	70.38%
POVH	76.09%

The proposed POVH provides discriminative spatial clues and has proven to be the best spatial feature extraction scheme by maintaining consistent performance on complex image benchmarks.

## 5 Discussion

The comprehensive evaluations on three challenging image benchmarks have demonstrated the effectiveness of the proposed approach for remote scene image classification. In this section, we provide a discussion on the feature dimensionality, sensitivity analysis and the limitations of the proposed research.

### 5.1 Feature dimensionality

To prove the computational efficiency of proposed approach, Table 6 provides a comparison with the state-of-the-art concurrent approaches. High-level approaches based on deep-learning outperformed the methods relying on mid-level representations [64]. Although deep-learning approaches have shown remarkable performance for large-scale context (1 million training examples), the significance of these approaches remains unclear for complex datasets with fewer training examples [65]. It is worth mentioning here that for relatively small datasets CNN-based approaches are not an optimal choice, as they require large-scale training data with a lot of computations to train a classification-based model [66]. It is established that deep-learning approaches are computationally expensive as they require huge amounts of data (in millions) and significant training time [24]. The BoVW model is a plug-n-play method which can be adopted without any prior initialization or training [56]. Hence for comparison, we selected the mid-level methods closely related to the proposed approach i.e. [24, 25].

**Table 6:** Comparison with closely related approaches using *N* visual words.

Scene Classification Method	Criteria of Comparison		
	Combinations of descriptors of *i*^*th*^ visual word	Dimensions of Histogram	Invariance to Rotation
PIWAH [24]	${}^{b_{i}}C_{2}$	9 × *N*	No
TIWAH [25]	${}^{b_{i}}C_{3}$	9 × *N*	Yes
POVH	${}^{b_{i}}C_{2}$	5 × *N*	Yes

From Table 6 we can see that both TIWAH [25] and POVH are invariant to rotation. An important factor influencing the computational complexity of the aforementioned approaches is the cardinality i.e. number of possible subsets of two distinct elements among *b*_*i*_ elements. Here, we compare POVH with relative spatial feature extraction approaches i.e. PIWAH [24] and TIWAH [25]. The possible number of combinations for TIWAH is ${}^{b_{i}}C_{3}$, whereas, for POVH and PIWAH the possible number of pair combinations are ${}^{b_{i}}C_{2}$. It means that for 50 identical visual words, the possible numbers of triplet combinations are 19600 and the possible pair combinations (for PIWAH and POVH) are 1225, which indicates that TIWAH is computationally expensive in terms of visual word combinations. It is important to mention here that the PIWAH approach is sensitive to rotation transformation. As the proposed approach is derived from PIWAH [24], it must be noted here that contrary to PIWAH [24], the proposed approach is invariant to rotation, which is a desired attribute for classification of remote sensing imagery. Here, we can see that for PIWAH [24] and TIWAH [25], the dimensions of resultant feature vector are 9 × *N*, where *N* is the size of visual vocabulary. For a visual vocabulary of size 200, the dimensions of PIWAH [24] and TIWAH [25] feature vector will be 1800, whereas, for POVH the dimensions of resultant feature vector will be 1000. The dimensions of POVH are $\frac{5}{9}$ of TIWAH and PIWAH respectively, which significantly increases the computational efficiency. Hence, it can be safely said that the POVH approach is computationally efficient as compared to the state-of-the-art relative spatial feature extraction approaches.

### 5.2 Sensitivity to rotation transformation

**Fig 12:**
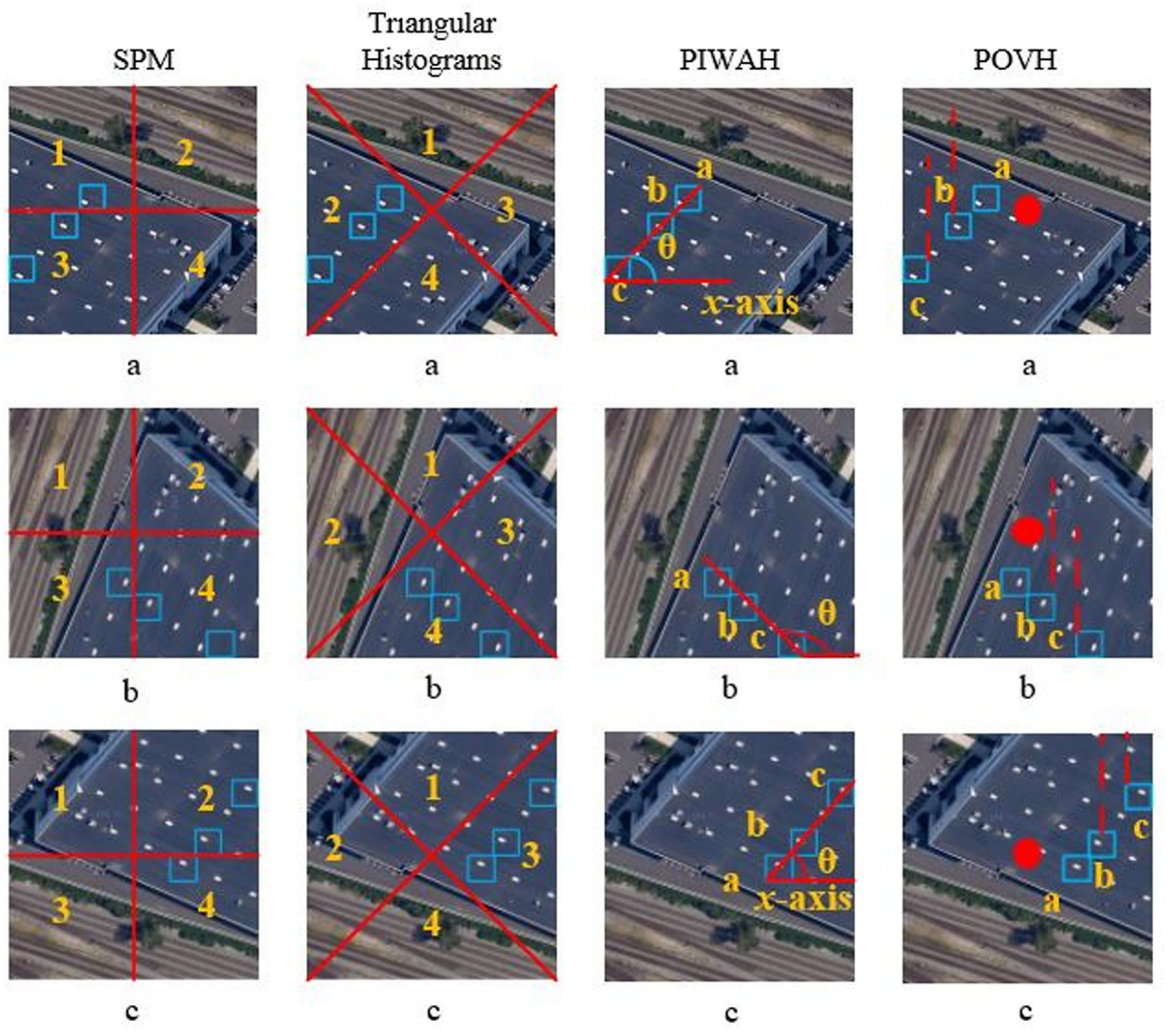
Image representation based on different spatial approaches where (a) represents the original image, (b) the image rotated by 90°, and (c) image rotated by 180°.

To illustrate the sensitivity of proposed approach to rotation invariance, Fig 12 provides a comparison of the proposed approach with the existing spatial feature extraction approaches. An important point of interest here is that the existing spatial feature extraction approaches are not appropriate for remote sensing scene classification. Lazebnik *et al*. [21] incorporated the spatial context to the BoVW model and achieved higher performance as compared to the traditional BoVW model. However, SPM is not invariant to basic transformations: such as rotation, as can be seen in Fig 12. Here a,b, and c illustrate; the original image, image rotated by 90° and the image rotated by 180° respectively. In Fig 12(a) for SPM, the visual words are located in regions 1 and 3 respectively, whereas, in Fig 12(b) they are found in sections 3 and 4. In the Fig 12(c) representation of same image, they can be seen in spatial regions 4 and 2 respectively. Hence, the histogram representation for the same image will be different, in each case.

In case of triangular histograms, the visual words in original image are in spatial region 2. While in rotated image (b), they are observed in region 4. In the 3^*rd*^ image representation, the visual words can be seen in the region 3 of spatial partitions. Hence it is evident, that the absolute spatial feature extraction approaches cannot handle challenging datasets that account for significant rotation differences. The experimental results presented in Section 4, demonstrate that BoVW without spatial information, outperforms these absolute spatial approaches, on remote sensing image datasets. Whereas, in literature, SPM and triangular histograms have demonstrated better performance on varying image benchmarks [16, 21]. The reason for this is quite obvious, as the aforementioned approaches can’t handle rotation of image, hence the performance degrades for high resolution remote sensing images.

Now, we provide a discussion on the relative spatial approaches i.e. PIWAH and the proposed POVH. It can be seen from Fig 12, that the angle computed between identical visual words is different for the original and the rotated images, as it is computed along x-axis. Whereas, in case of the proposed approach the orthogonal vectors for the identical visual word pairs are calculated relative to the geometric center of an image. POVH not only enhances the discriminative power of model by adding information regarding collinear points in an image, but also makes the proposed approach invariant to basic transformations: such as rotation and flipping. It is evident from Fig 12, that the magnitude of orthogonal vector remains same for the original (a) and the rotated images (b and c). The proposed research outperforms the other image spatial representations in terms of classification accuracy.

It must be noted that numerous techniques have been proposed in literature [67], that involve the fusion of SPM with other features and techniques to enhance the classification performance. The remarkable performance of the proposed approach makes it a potential candidate to be used in combination with other approaches. It would be interesting to observe the outcomes of the proposed approach in fusion with other techniques, to boost the classification performance.

### 5.3 Limitations and future work

Our proposed approach provides outstanding results on remote sensing image benchmarks and outperforms the state-of-the-art spatial feature extraction approaches. However, there are some also some open issues to be addressed in future research. For example, the recent research trend is directed towards the application of deep neural networks due to its strong feature representation powers and higher performance for large-scale image classification [47]. We intend to develop a framework, to extract features by applying some deep-learning techniques and extract the relative spatial information using the proposed POVH approach. SVM requires a substantial number of training images whereas deep-learning approaches provide better outcomes even with low number of training images. For our experiments, we followed the training test ratio 0.7:0.3 for all datasets. We intend to optimize our approach to provide promising results even with fewer training samples.

The major contribution of the proposed research is the addition of spatial information to the BoVW model in a rotation-invariant manner. The spatial clues are extremely important for remote sensing scene classification. This is the reason, many deep-learning approaches use SPM to incorporate the spatial context in their implementation [68, 69]. The experimental results presented in Section 4, prove that our proposed spatial feature extraction technique outperforms the state-of-the-art spatial feature extraction approaches. In our contribution, we suggest that when we have features that are human interpretable, it is much easier to understand the cause of its decision. In future, we intend to compare the handcrafted methods with the machine-crafted ones (pre-trained and non-pre-trained networks). A direct extension of this work is to create a hybrid approach, by fusion of the proposed spatial feature extraction technique with the deep-learning approaches to enhance the classification performance. The proposed approach provides complementary information to the global correspondence methods as SPM [21] and triangular histograms [16]. A fusion of absolute [16, 21] and the proposed relative method would be promising direction for future research. In addition to this, the ability to integrate local spatial information, and the spatial information provided by global cues such as color, to this framework is an open area of research.

## 6 Conclusions

In this paper, we propose a novel approach that incorporates the global relative spatial information to the inverted index of the BoVW model. This is done by computing histograms based on the magnitude of orthogonal vectors between PIVW relative to the geometric center of an image. A comparative analysis is performed with the state-of-the-art spatial feature extraction approaches to obtain the best representation. The research presented in this paper is evaluated by using three challenging image benchmarks of remote sensing.

The proposed research outperforms the existing state-of-the art approaches in terms of classification accuracy. The proposed approach provides discriminative features and invariance to geometrical changes in the remote sensing images. Extensive comparisons on challenging remote sensing image benchmarks validate the effectiveness of the proposed POVH for remotely sensed land-use scene classification. In future, we aim to extend our research by using a pre-trained deep neural network for histogram computation to train classifier for a large scale image dataset.
